# Worker studies and their interpretation

**DOI:** 10.1088/1361-6498/ade68e

**Published:** 2025-07-14

**Authors:** David B Richardson, Dominique Laurier, Richard Haylock, Kaitlin Kelly-Reif, Stephen Bertke, Robert D Daniels, Isabelle Thierry-Chef, Ausrele Kesminiene, Mary K Schubauer-Berigan

**Affiliations:** 1 Department of Environmental and Occupational Health, Program in Public Health, University of California, Irvine, CA 92697, United States of America; 2 Institut de Radioprotection et de Sûreté Nucléaire (IRSN), PSE-SANTE, F-92260 Fontenay-aux-Roses, France; 3 UK Health Security Agency, Chilton, Didcot, Oxfordshire OX110RQ, United Kingdom; 4 National Institute for Occupational Safety and Health, Cincinnati, OH, United States of America; 5 Barcelona Institute of Global Health (ISGlobal), Barcelona, Spain; 6 International Agency for Research on Cancer., Lyon, France

**Keywords:** workers, interpretation, cohort studies, mortality, cancer, epidemiology

## Abstract

A recent commentary on epidemiological studies of nuclear workers notes that these studies can provide radiation risk estimates that complement those derived from the study of Japanese atomic bomb survivors. The author asserts that the results from some nuclear worker studies are difficult to interpret due to the fact that ERR/Gy estimates vary across subcohorts, and subcohort-specific estimates are not always equal to estimates obtained in the overall study population. We discuss settings in which it is reasonable to expect that an estimate of association in a subcohort should be similar to an estimate obtained in the full cohort and settings in which a subcohort analysis may differ from the estimate obtained in a full cohort analysis. Focusing on the INWORKS study, we describe some of the steps taken to understand variation in estimates of ERR/Gy between subgroups and upon restrictions, as well as interpretation of estimates of external dose-mortality associations in the total study population.

Professor Wakeford’s recent commentary [1] on epidemiological studies of nuclear workers notes that these studies can provide radiation risk estimates that complement those derived from the study of Japanese atomic bomb survivors, given that workers experience protracted exposures to radiation whereas the atomic bomb survivors experienced an acute high dose rate exposure. However, Wakeford asserts that the results from some nuclear worker studies, notably INWORKS, are ‘disappointingly difficult’ to interpret [1].

His comments focus primarily on the fact that ERR/Gy estimates vary across subcohorts, and subcohort-specific estimates are not always consistent with estimates obtained in the overall study population.

## 1. Should we expect an ERR/Gy to be invariant?

If a subcohort is a random sample of the full study cohort, then it is reasonable to expect that an estimate of association in the subcohort should be similar to an estimate obtained in the full cohort. However, Wakeford discusses subcohorts that are not random samples of the full cohort. A summary estimate of the association between external radiation dose and cancer will differ in a subcohort analysis from the estimate obtained in a full cohort analysis when factors that modify the association differ between the subcohort and full cohort. For example, in INWORKS, subcohorts that are defined by year of hire differ markedly from each other, and from the full study cohort, with respect to time-since-first-exposure, attained age, birth cohort, duration of follow-up, and other factors that exhibit secular trends. When factors, such as these, modify the ERR/Gy, we should expect the ERR/Gy to vary across subcohorts.

We are certainly interested in what explains variations in estimates of ERR/Gy between subgroups and upon restrictions. However, we emphasise two points:

i) Modifiers of radiation-associated cancer risks are difficult to reliably estimate with precision in low dose studies; and,
ii) Marginal estimates of radiation risk are informative because a summary ERR/Gy estimate describes the average effect of exposure on cancer in the study cohort, which is a target population of interest.

## 2. What do we know about the reasons for variation?

We considered factors that could lead to such variation in estimates of ERR/Gy across subcohorts of INWORKS.

• Some factors that could lead to variations in ERR/Gy estimates between subcohorts relate to measurement error. Exposure measurement errors may be larger in the early years of the nuclear industry than later, and could lead to greater bias in estimates of ERR/Gy for subcohorts of early workers than subcohorts of workers hired later. Such an impact has been observed, for example, in the assessment of the lung cancer risk related to radon exposure in uranium miner studies [2]. However, if exposure measurement error was the reason for variation by period of hire in ERR/Gy for solid cancer in INWORKS, then it also would be expected to lead to variation by period of hire in ERR/Gy for leukaemia; there was minimal variation by period of hire in the radiation dose-leukaemia mortality association in INWORKS [3].
• For this study, correction factors were applied to estimate organ absorbed dose from the facility-recorded ‘whole-body’ dose values. These correction factors were derived to account for exposure geometry, body attenuation, and differences in dosimetry methods between facilities and over time [4]. Although temporal differences in dose correction were noted, the variation in ERR/Gy by hire period was not explained by these differences; analyses based on recorded doses (i.e. the facility reported dose values) yielded similar patterns to analyses based on our estimates of organ absorbed dose.
• Temporal factors, such as time since exposure or attained age, could lead to variations in association between subcohorts. Such an effect has been observed in other occupational studies [5]. Although we noted an older average attained age and a longer average time since exposure among early workers, we have not seen strong evidence of modification of the ERR/Gy by these temporal factors in INWORKS [6].
• Dose distributions differ between subcohorts [1, 7–9]. However, attenuation of the dose-response at high cumulative dose among early workers does not appear to explain variation in ERR/Gy by hire period in INWORKS; estimates of ERR/Gy among early hires exhibit minimal downward curvature at high dose.

More compelling is evidence of submultiplicative interaction of dose with birth cohort, and with factors such as smoking that are correlated with it. Submultiplicative interaction between radiation and smoking has been reported in other radiation-exposed cohorts [10–13]. In INWORKS, we do not have individual smoking histories; however, we observe that birth and hire cohort are highly correlated, and we observe substantial variation in baseline cancer rates by birth cohort, which conforms with population-level changes in smoking across birth cohorts. When subcohorts differ with respect to factors that modify the ERR/Gy, such as birth cohort (or factors associated with birth cohort), we expect the ERR/Gy to vary across these subcohorts. Further work is ongoing to investigate this point in INWORKS.

## 3. Conclusion

INWORKS analyses have focused on the estimation of external dose-mortality associations in the total study population [14]. This yields estimates of ERR/Gy that are marginal over the distribution of effect measure modifiers in the total study cohort. There is a useful interpretation of such summary ERR/Gy estimate from INWORKS: it provides a summary estimate of the association between cumulative radiation dose and cancer mortality in a study population of historical nuclear workers who were exposed to low dose-rate radiation throughout their working lives.

Many researchers involved in nuclear worker studies have dedicated, and will continue to dedicate, time and attention to understanding the results of these studies, including research to understand factors that influence baseline mortality rates, exposure measurement errors, confounding and selection bias. Building upon those efforts, recent reports on nuclear worker cohort studies yield highly informative estimates of radiation-cancer associations that provide an important complement to the evidence of radiation-cancer associations derived from the Life Span Study of Japanese atomic bomb survivors and other radiation-exposed populations [15–17]. It is important to continue to investigate whether observed associations are biased. However, the notion that a given measure of association should *a priori* be invariant across subcohorts, and when not that this indicates bias, is inconsistent with contemporary thinking on causation and transportability. Subcohort analyses are seldom the best strategy for assessing bias in epidemiological studies. Restriction can induce bias, and restriction often will lead to a change in a measure of association from that observed in the full cohort simply because of differences in distributions in the effect measure modifiers [18, 19]. There are far better approaches to assess bias [20, 21]. We thank Wakeford [1] for his commentary which encourages reflection on the important contributions of several large occupational cohort analyses of nuclear workers.

## Data Availability

The data that support the findings of this study are available upon reasonable request from the authors.

## Disclaimer

The findings and conclusions in this report are those of the authors and do not necessarily represent the official position of the National Institute for Occupational Safety and Health, Centers for Disease Control and Prevention. Where authors are identified as personnel of the International Agency for Research on Cancer/World Health Organization, the authors alone are responsible for the views expressed in this article and they do not necessarily represent the decisions, policy or views of the International Agency for Research on Cancer/World Health Organization.
